# A systematic review of multimodal machine learning models for heart failure classification and prognosis prediction

**DOI:** 10.3389/fcvm.2026.1770869

**Published:** 2026-04-15

**Authors:** Manh Thang Hoang, Yang Chen, Gregory Slabaugh, Nay Aung

**Affiliations:** 1 William Harvey Research Institute, Queen Mary University of London, Charterhouse Square, London, United Kingdom; 2 Digital Environment Research Institute (DERI), Queen Mary University of London, London, United Kingdom; 3 NIHR Barts Cardiovascular Biomedical Research Centre, Queen Mary University of London, Charterhouse Square, London, United Kingdom; 4 Barts Heart Centre, Barts Health NHS Trust, West Smithfield, London, United Kingdom

**Keywords:** heart failure, machine learning, meta analysis, multimodal, review

## Abstract

**Introduction:**

Heart failure (HF) is a global medical condition marked by substantial morbidity, mortality, and healthcare costs with complex pathophysiology and variation in definitions. Machine learning (ML) has emerged as a promising approach to improve HF classification and risk prediction by leveraging various data sources. This study aims to present the current state-of-the-art multimodal ML models for HF classification and prognosis prediction, focusing on their modalities, performance, and clinical utility.

**Methods:**

Following PRISMA guidelines and registered with PROSPERO (CRD420250654631), this review searched across four electronic databases (November 2014 – November 2024) and identified 284 unique records, of which 15 were included in the final synthesis. The quality of the studies was evaluated using QUADAS-2 and QUAPAS.

**Results:**

Our results showed that the two most common multimodal combinations were tabular-image and tabular-text. The algorithms of the models included convolutional neural networks for image data, transformer-based approaches for text, with well-known fused techniques (early, middle, late fusion). Overall, multimodal models demonstrated superior performance compared to unimodal approaches, achieving area under the receiver operating characteristic curve values frequently exceeding 80% and reaching as high as 98.2%.

**Conclusion:**

Despite promising results, challenges include inconsistent reporting of performance metrics and their 95% confidence intervals, limited external validation, a near absence of prospective studies, and a deficiency in integrating genetic or 'omics' information with conventional data. These challenges must be addressed to promote clinical adoption and future research

**Systematic Review Registration:**

https://www.crd.york.ac.uk/PROSPERO/view/CRD420250654631, identifier CRD420250654631.

## 1 Introduction

Heart failure (HF) is a medical condition that occurs when the heart struggles to either pump blood efficiently or fill it sufficiently (1). It is a major disease that is considered a global pandemic, with substantial morbidity, mortality, and healthcare expenditures (1). Approximately 56.2 million people were living with HF worldwide in 2019 (2). In the UK, HF accounts for a significant portion of hospital admissions, and projections indicate a more than 50% increase in hospitalisations by 2035, which is partly attributable to an ageing population (3). Because of the multitude of aetiologies that underlie its pathophysiology, HF is more complex than diseases having a pathologic gold standard for diagnosis (4). Therefore, there is considerable variation in the definitions of HF in the medical literature, practice, and guidelines (4). In addition to that, conventional diagnosis based on the left ventricular ejection fraction (4–6) often fails to capture the nuanced complexity of HF pathophysiology and individual patient trajectories (4).

Machine learning (ML) has emerged as a promising approach to improve HF classification and risk prediction, utilising various data sources such as genetic information and electronic health records (EHRs), offering new avenues for personalised HF management (7–9). By leveraging multiple data types—including clinical records, imaging, “omics” data, and physiological signals—a multimodal ML model has the potential to uncover novel insights potentially missed by analysing only single data type (10). To optimally integrate this heterogeneous information for improved performance, researchers have developed approaches to fuse data types at different stage of their models. These approaches can be broadly classified as early, middle, or late fusion with distinct advantages and challenges. Early fusion combines raw features from all modalities upfront, allowing models to learn from the collective low-level data but sometimes obscuring inter-modality relationships or hindering the feasibility if input data formats are extremely different. Middle fusion extracts features from each modality independently, then integrates them—often with neural network architectures or transformer-based attention mechanisms—to capture nuanced, cross-modal interactions. Late fusion merges the outputs or decisions of separate models trained on each data source, leveraging the complementary strengths of different modalities, but may overlook complex shared patterns (11, 12).

However, the applicability of these multimodal ML models in clinical practice remains unproven. The challenges include, but are not limited to, internal validity and external generalisability (13). To date, we cannot find a comprehensive systematic review, which is either in literature databases or the Prospective Register of Systematic Reviews (PROSPERO)—an international systematic review registry (14), that has critically evaluated the potential of multimodal ML models for both HF classification and prognosis prediction.

This study conducts a systematic literature review on multimodal ML models for HF classification and its prognosis prediction. Our aims are as follows: 1) to present the current landscape of multimodal ML approaches for HF classification and its prognosis prediction; 2) to summarise the datasets, input modalities, data fusion techniques and algorithms used in the published multimodal ML models; and 3) to report the models' performance and their clinical utility. By systematically examining ML techniques across diverse data modalities, we seek to provide insights into the potential transformative role of integrating various data sources in HF diagnosis and prognostication.

## 2 Materials

We followed the Preferred Reporting Items for Systematic reviews and Meta-Analysis (PRISMA) guidelines in this systematic review. Our study assessed and synthesised publicly available studies; therefore, the ethics committee was waived. The present study was registered with PROSPERO (PROSPERO ID: CRD420250654631).

### 2.1 Search strategy

Four electronic databases, the Web of Science Core Collection, Embase, PubMed, and the IEEE Xplore Library, were searched by a reviewer (M.T.). The search date was 7 November 2024, with the publication date restricted to the past 10 years. Titles and abstracts were searched via the keywords presented in the supplement. In brief, the keywords used in all the databases pertained to HF, ML, multimodality, and classification or prognosis.

### 2.2 Inclusion and exclusion criteria

To enhance the comprehensiveness of the search, the criteria for inclusion were deliberately made wide-ranging. This study aimed to identify the articles that applied any multimodal ML model for HF classification in study participants and HF prognosis among known HF patients. Table 1 shows the additional included and excluded criteria.

**Table 1:** Inclusion and exclusion criteria used in the review.

Type	Inclusion	Exclusion
Study design	Retrospective, prospective cohorts	None
	Case-control studies Randomized control trials	
Population		
Classification objective	HF patients with/without non-HF patients	None
Prognosis objective	HF patients	None
Intervention	Any ML models	Single-modality methods
Exposure	All	None
Reported outcomes	At least one of precision, recall, accuracy, F1-score, area under the receiver operating characteristic curve	No ML metric reported
Language	English	Any other language
Geographic location of study	All	None

HF, Heart failure; ML, Machine learning.

After conducting the electronic searches, we imported all the retrieved titles and abstracts into Rayyan (15) and deleted duplicates. Full papers of studies meeting the inclusion criteria based on initial title/abstract screening were then collected for in-depth inspection. Two reviewers (M.T. and Y.C.) independently evaluated the retrieved studies' eligibility and discussed any disagreements together or with a third review author (N.A.).

### 2.3 Data extraction

Regarding the evaluation of study quality and the evidence's synthesis, a standardised data extraction sheet, mutually accepted by the coauthors, was employed to collect relevant information from the qualifying studies. The extracted information, done by two reviewers (M.T. and Y.C.), included Author, Publish Date and Publisher, Objective (classification or prognosis prediction task), Dataset, Training set, Test set, Internal or External set, Modalities used for models, Fusion strategy, Model architecture, and Model performance. Regarding the fusion strategy, we mainly recorded their technique as early, middle, and late fusion to facilitate better comparison among the ML models (11).

### 2.4 Risk of bias assessment

Two reviewers (M.T. and Y.C.) independently assessed the quality (risk of bias and applicability concerns) using QUADAS-2 (16) for studies with a classification objective and QUAPAS (17) for studies with a prognosis prediction objective. Disagreements were resolved through discussion together or with a third reviewer (N.A.).

## 3 Results

### 3.1 Study selection

The initial search identified 284 unique records after duplicate removal. After applying the included and excluded criteria (Table 1), title and abstract screening eliminated 237 records. Of the remaining 47 full-text publications assessed for eligibility, 32 were subsequently excluded, resulting in 15 studies meeting all the inclusion criteria. Among them, ten studies aimed at HF classification, four studies at HF prognosis prediction, and one at both objectives. The included studies' characteristics are presented in Table 2.

**Table 2:** General characteristics and datasets used of the included studies.

Author	Publish Year, Publisher	Objective	Dataset	Training	Test set	I/E
Vahabi N et al. (50)	2021, Frontiers in Genetics	P	91 HF patients (both preserved and reduced ejection fraction; cut points not reported) attending cardiology clinics at the University of Illinois at Chicago between 2011 to 2015, consisting of 26,379 mRNA expression, 578,856 genotype and 12,283 DNA methylation profiles	80% of original data	20% of original data	I hold out
Ma Huiting et al. (58)	2024, Information Systems	P	9,789 HF patients (ICD-9-CM codes) derived from MIMIC-III database	8,810 patients	979 patients	I hold out
				Training/validation: 8/2		
Zhenyue Gao et al. (59)	2024, Journal of medical Internet research	P	21,814 HF patients derived from − MIMIC-III (ICD-9 codes; development and internal validation) − MIMIC-IV v1.0 (ICD-10 codes; development, internal and prospective validation) − eICU Collaborative Research Database v1.2 (ICD-10 codes; external validation)	9,989 patients	Internal test set: 2,497 patients	I and E
					Prospective test set: 1,896 patients	
					External test set: 7,432 patients	
Ma Meikun et al. (63)	2023, Medical & biological engineering & computing	P	6,296 HF subjects (ICD-9 code 4280) from MIMIC-III equivalent to 18,464 EHR samples	80% of original data	20% of original data	I hold out
				Training/validation: 3/1		
González Sergio et al. (60)	2014, Information Fusion	P and C	24,125 subjects from three datasets - Holter ECG: 21,891 HF patients (primary discharge diagnosis) - MIMIC-III: 2,201 subjects (9.6% HF; ICD-9 codes) - PhysioBank: 33 subjects (45.5% severe congestive HF)	70% of Holter ECG dataset	Internal test: 30% of Holter ECG dataset	I and E
				Training/validation: 8/2	External test: MIMIC-III, PhysioBank	
Farajidavar N et al. (52)	2022, BMC Cardiovascular Disorders	C	1,854 consecutive patients attending King's College Hospital NHS Foundation Trust in London between 2000 and 2019. HFpEF: LVEF ≥50% (never <50%), ICD-10 codes I50.0/I50.1/I50.9, dyspnea, and elevated NT-proBNP per ESC criteria. Controls: non-HF (no HF diagnosis, dyspnea, elevated BNP, or reduced LVEF) and HFrEF (HF diagnosis with LVEF <50%)	1,585 patients	269 patients	I hold out
				Training/validation: 8/2		
Liu Yi et al. (53)	2023, Multimedia Tools and Applications	C	440 patients attending the Shanxi Cardiovascular Disease Hospital: 325 HF patients without diabetic complications (specific HF definition not reported) and 115 mild CVD patients	80% of original data	20% of original data	I hold out
Botros Jad et al. (57)	2024, Biomedical Signal Processing and Control	C	2,500 people derived from MIMIC-IV database: 1,250 HF patients (HF definition not reported) and 1,250 controls (healthy individuals or patients with other diseases)	Training/validation: 1,500/500 people	500 people	I hold out
Postiglione Marco et al. (54)	2024, Image Analysis and Processing—ICIAP 2023 Workshops	C	54,462 patients (HF and other diseases; HF physician-diagnosed) attending the Hospital of Naples Federico II	80% of original data	20% of original data	I hold out
Shiraga Takeru et al. (55)	2023, Sensors	C	1,155 patients attending Division of Cardiology, Medical Faculty, Heinrich-Heine-University Düsseldorf. HF defined as LVEF ≤ 40%	1,052 patients	103 patients from an independent cohort	I hold out
				Training/validation: 839/213 (∼8/2)		
Hardy-Werbin Max et al. (56)	2023, Scientific reports	C	6,123 patients and healthy subjects from Parc Salut Mar Consortium, Barcelona with 8,578 entries of paired blood test and CXR data, including 1,008 from patients with HF episodes (2012–2019)	7,755 paired entries	823 entries for experiment and 300 random test images to check with five experienced thoracic radiologists	I hold out
				80% training, 20% validation		
Ketabi Sara et al. (61)	2023, MICCAI Workshops 2023	C	1,017 patients (congestive HF and other diseases; congestive HF ICD-9 codes) from MIMIC-CXR database including 1,017 paired x-ray images and radiology reports	80% of original data	20% of original data	I hold out
Zhang Shenghan et al. (62)	2023, IEEE 11th International Conference on Healthcare Informatics	C	30,601 subjects from MIMIC-III including 10,109 HF patients (identified using validated PheKB algorithm combining ICD codes and echocardiogram results) and 10,109 paired negatived subjects	80% of original data	20% of original data	I hold out
Lu Yuan et al. (51)	2024, 16th International Conference on Intelligent Human-Machine Systems and Cybernetics	C	MIMIC-CXR (specific HF definition not reported)	Training: 12,896 CXR-Clinical Text pairs	1,612 CXR-Clinical Text pairs	I hold out
				Validation: 1,612 CXR-Clinical Text pairs		
Lee Chih-Kuo et al. (64)	2024, Computer Methods and Programs in Biomedicine	C	1,432 suspected acute HF patients from MIMIC-IV, MIMIC-CXR, and JSRT equivalent to 1,833 pairs of CXRs and EHRs. Later, acute HF diagnosis (model outcome) defined as NT-proBNP > 300 ng/L	1,464 pairs of CXRs and EHRs	369 pairs of CXRs and EHRs	I hold out

C, Classification objective; CVD, Cardiovascular disease; CXR, Chest x-ray; ECG, Electrocardiogram; EHR, Electronic health record; HF, Heart failure; HFpEF, Heart failure preserved ejection fraction; HFrEF, Heart failure reduced ejection fraction; I/E, Internal/External; LVEF, Left ventricular ejection fraction; NT-proBNP, N-terminal pro-brain natriuretic peptide; P, Prognosis objective.

**Figure 1:**
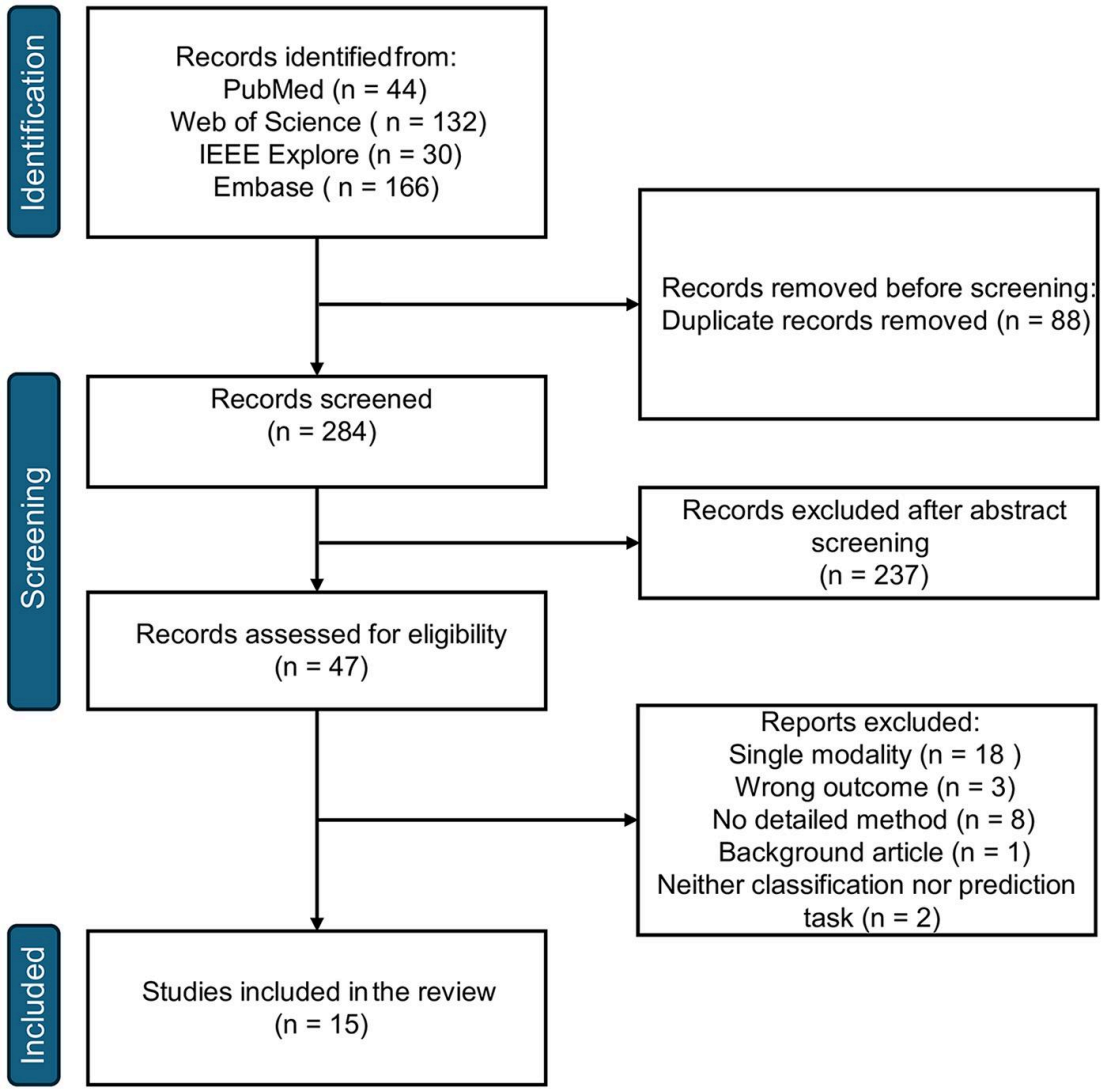
Prisma flow diagram of the included studies.

The reasons for exclusion were as follows: 18 studies employed single-modality ML approaches (18–35); eight studies provided insufficient details on ML architecture (36–43); three studies investigated general cardiovascular or heart diseases without specifically addressing HF outcomes (44–46); two studies either utilised epidemiological approaches with singular input data type (47) or examined heart rate variability as an autonomic nervous system function measurement in congestive HF patients with severe arrhythmia (48); and one background study merely described the UK Heart Failure with Preserved Ejection Fraction Registry profile (49). Figure 1 illustrates the process of article selection for this systematic review.

**Figure 2:**
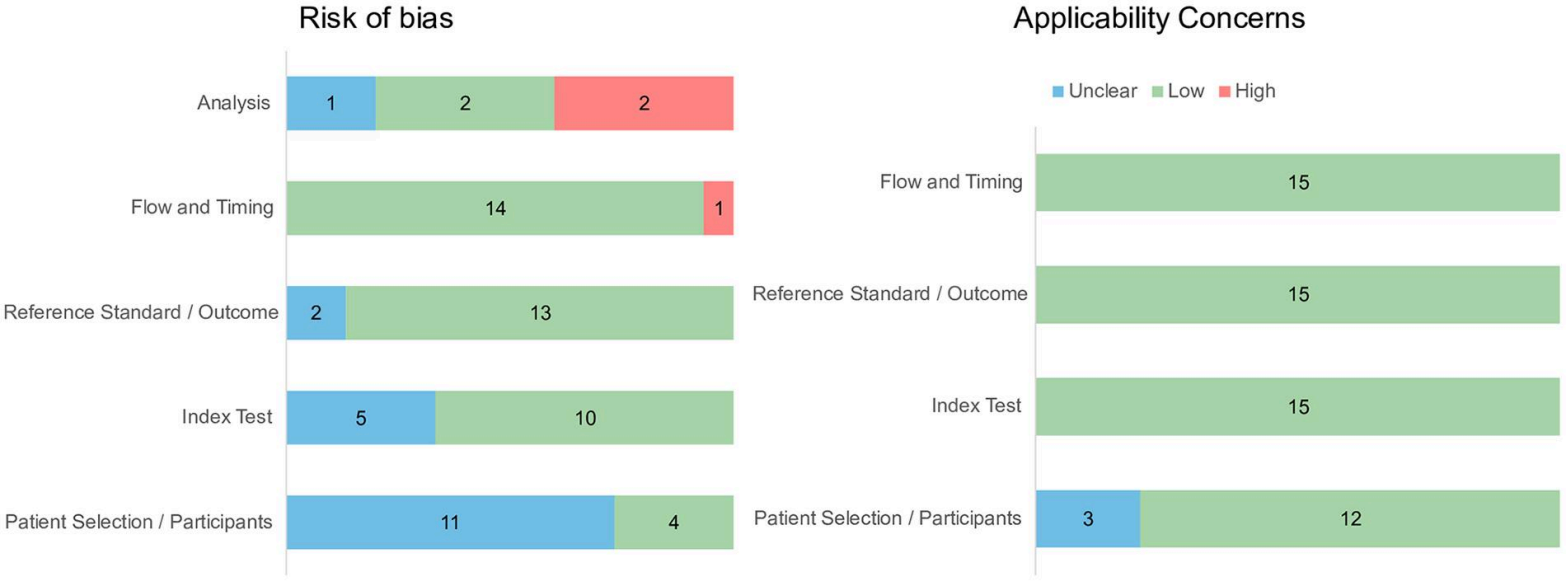
Quality assessment for 15 included studies based on QUADAS-2 and QUAPAS.

We found two studies (prognosis prediction objective) with a high risk of bias in the analysis domain and one study (classification objective) with a high risk of bias in the flow and timing domain. All included studies had low or uncertain concerns of applicability. Details of the QUADAS-2 and QUAPAS scores for each study are demonstrated in Figure 2.

**Figure 3:**
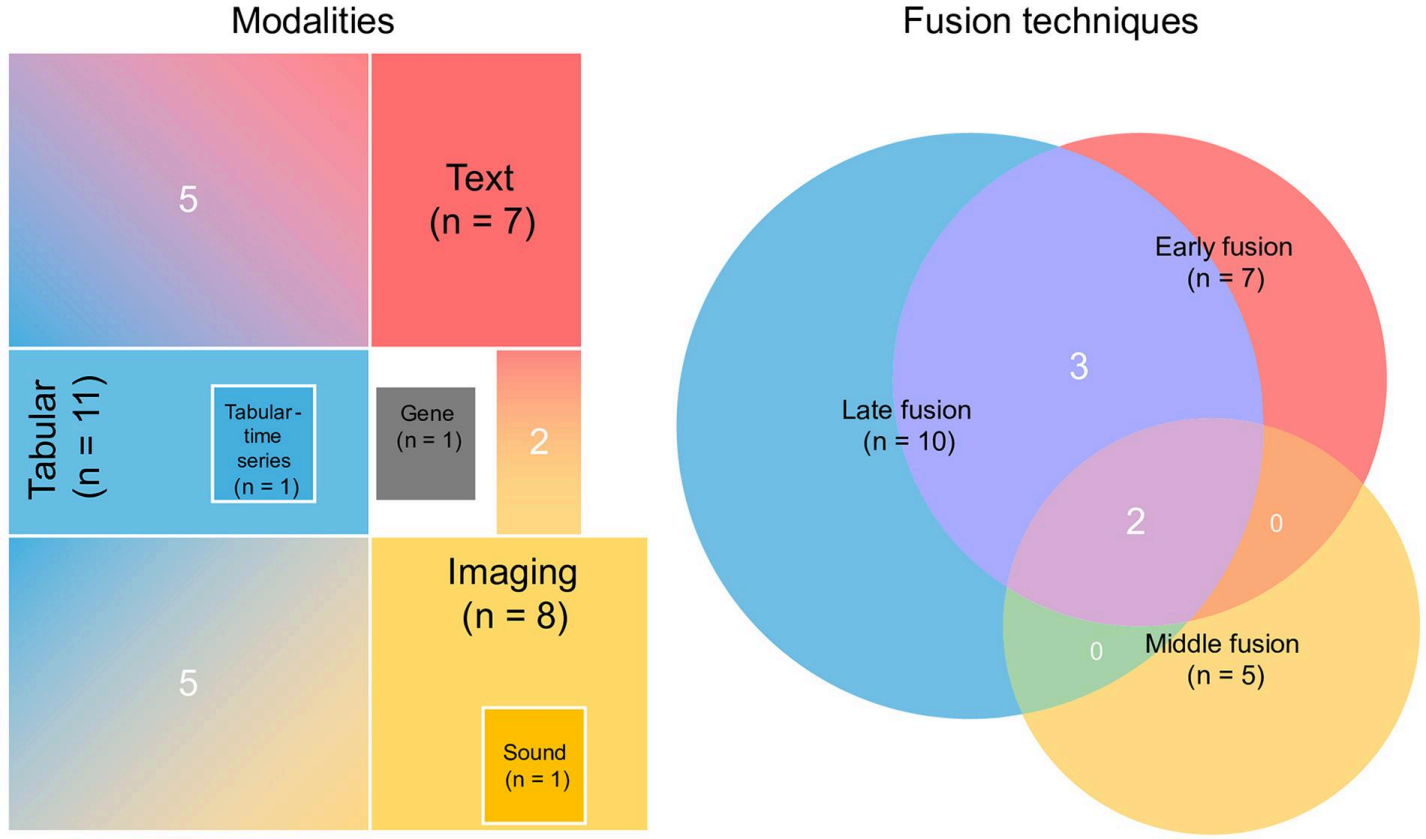
Number of studies utilising different modalities and fusion techniques.

### 3.2 Databases

Regarding sample sizes, one study (50) (6.7%) utilised genetic data with fewer than 100 individuals, nine studies (60%) included between 100 and 1,000 individuals, four studies (26.7%) included approximately 21,000 to 55,000 individuals, and one study (51) (6.7%) omitted sample size reporting. Six studies employed institutional/hospital datasets (50, 52–56). Moreover, the remaining studies utilised publicly available repositories: nine studies (51, 57–64) incorporated the MIMIC database and its variants, one study (59) leveraged the eICU Collaborative Research Database, one study (60) utilised Physio Bank and Holter Electrocardiogram (ECG) datasets, and one study (64) employed the JSRT database (the standard digital image database of chest radiographs with and without lung nodules from the Japanese Society of Radiological Technology).

### 3.3 Modalities and fusion techniques

Among the 15 included studies, tabular features constituted the predominant modality, appearing in 11 studies (73.3%), followed by imaging features derived primarily from ECGs and chest x-rays in eight studies (Figure 3). Only one study incorporated genetic features as model inputs (50). The most prevalent multimodal combinations were tabular-image and tabular-text (both 41.7%), followed by text-image (16.7%), tabular-time series data (8.3%), image-sound (8.3%) and multi-omics (8.3%). We did not find any studies that combined genetic modalities with other modalities or that integrated three or more modalities.

Regarding fusion strategies, majority of studies employed late fusion technique (66.7%), followed by early fusion technique (46.7%) and middle fusion technique (33.3%). Two studies compared all three fusion approaches, with late fusion demonstrating superior performance for classification tasks (57, 64). Similarly, a different study using early and late fusion techniques for prognosis prediction tasks indicated that late fusion performed better (60). This tendency may reflect not only the advantage of modality-specific feature extraction before integration, but also the inherent limitations of alternative strategies. Early fusion concatenates raw features at the input level; however, aligning heterogeneous data types with differing scales risks one dominant modality overshadowing others. Middle fusion integrates intermediate representations, requiring larger datasets to learn meaningful cross-modal interactions—a notable constraint given the limited sample sizes observed in this review. Late fusion, while straightforward to implement, assumes conditional independence among modalities, which may not hold where ECG abnormalities and imaging findings are patho-physiologically correlated. In addition to these three fundamental fusion approaches, several researchers have incorporated sophisticated integration strategies (51, 58, 59, 62, 63), including attention mechanisms (51, 59) and graph-based structures (58).

### 3.4 Algorithms

Table 3 indicates that seven studies implemented convolutional neural networks for image data processing (51, 53, 55, 57, 60, 61, 64), with DenseNet appearing in two studies (53, 64), ResNet variants in two studies (51, 60), and EfficientNet in one study (61). For clinical text processing, transformer-based approaches predominated, with three studies employing BERT variants (51, 59, 62). Overall, transfer learning approaches—leveraging pretrained architectures for both image and text modalities—were utilised in six studies (51, 53, 59, 61, 62, 64). Traditional ML methods were primarily used for tabular data, with six studies implementing ensemble approaches (52, 54, 56, 57, 62, 64), including XGBoost (52, 57) and Random Forest (54). Furthermore, some specialised components addressing healthcare-specific challenges were incorporated. They included a squeeze-and-excitation block with transformers for temporal ECG analysis (60), missing data imputation via variational recurrent neural networks (63), and multiblock partial least squares for high-dimensional data (50). The architectural complexity across studies typically matches the heterogeneity of the input data types being integrated, with studies employing distinct network components for different modalities before fusion.

**Table 3:** Tasks, modalities and performance metrics of the included studies.

Author	Objective	Outcome	Modalities	Fusion strategy	Model Architecture	Best model performance metrics (95% CI)^a^
Vahabi N et al. (50)	P	Reweighted survival time	- mRNA expression - Genotype - DNA methylation	Middle fusion	Supervised Cox sparse Multi-Block Partial Least Squares	Harrell's C-index 0.75
						AUROC 97.3
						Uno's AUROC 91.1
Ma Huiting et al. (58)	P	Multi-class classification task: no readmission, readmission within and after 30 days	- Tabular features (structured clinical features from EHR) - Text features (unstructured clinical records in EHR)	Dynamic middle fusion	For structured data, authors suggested an enhanced Deep&Cross Network to automate feature selection during encoding process.	The MDL-HFP model achieved macro-AUROC of 95.4, F1 score of 90.42, accuracy of 90.70, precision of 91.16, recall of 90.80
					For unstructured data, authors used an implicit graph structure information derived from the HR-BGCN model and paragraph topic similarity to weakly guide the graph attention mechanism	
Zhenyue Gao et al. (59)	P	Binary classification task: survival or not	- Tabular features - Text features (admission clinical notes)	Late fusion	A pretrained Bidirectional Encoder Representation from Transformers was used to process and extract feature from text input.	Text and tabular multimodal attention model achieved performance in - Internal test set (95% CI): AUROC of 83.9 (82.7–85.2), F1 score of 46.9 (43.0–51.0), AUPRC of 29.3 (26.4–32.1) - Prospective test set: AUROC of 84.9 (84.1–85.7), F1 score of 48.8 (47.5–50.1), AUPRC of 31.2 (30.1–32.4) - External test set: AUROC of 76.7 (76.2–77.2), F1 score of 41.5 (40.8–42.1), AUPRC of 25.2 (24.6–25.6)
					A gate attention mechanism combined the extracted text features and embedded tabular features	
Ma Meikun et al. (63)	P	Binary classification task: survival or not in 30 days after a fixed observation window (5, 7 or 10 days)	- Tabular features (categories features) - Tabular time series features (laboratory longitudinal measurements)	Middle fusion	The deep fusion learning-IMP model was constructed of three primary elements: GRU-S, VRNN missing value imputation, and mortality prediction of feature fusion.	Using a 5-day observation period and a 30-day prediction timeframe, the proposed deep fusion learning-IMP model with fusion strategy gave AUROC of 91.4 (91.33–91.47), F1 score of 73.4 (73.31–73.49), Accuracy of 92.8 (92.79–92.81), Precision of 86.7 (86.59–86.80), Specificity of 98.2 (98.19–98.21)
González Sergio et al. (60)	P and C	Prognosis objective: survival time	- Tabular data (demographic, history conditions) - Imaging features (ECG, sampled long term HRV)	Early fusion for XGBoost	The foundational architecture was a ResNet model designed to learn directly from unprocessed ECG signals. This base model featured an initial extraction layer and was followed by four residual blocks. Each of these residual blocks contained two layers of 1D CNN, batch normalization, and a ReLU activation function, succeeded by a Squeeze-and-Excitation block. To harness the temporal relationships within the HRV series, the researchers augmented this ResNet structure with a Transformer module.	Performance of survival model (TFM-ResNet) on the internal test set were: AUROC of 86.88, C-index of 0.8537, c/d AUROC of 87.18, iBS of 0.0744 Performance of classification model (TFM-ResNet) on the external test set - MIMIC-III: AUROC of 81.52, average precision of 35.99, recall of 73.33, specificity of 78.38, G-mean of 0.7581 - PhysioBank: AUROC of 95.84, average precision of 95.22, recall of 94.44, specificity of 82.58, G-mean of 0.8831
		Classification objective: Binary classification task: HF and non-HF		Late fusion for TFM-ResNet Model		
Farajidavar N et al. (52)	C	Averaging signature likelihood predictions of two binary classification tasks: HF reduced ejection fraction versus HF preserved ejection fraction and non-HF versus HF preserved ejection fraction	- Tabular features (structured EHR for clinical and demographic data, laboratory and echocardiographic measurements) - Text features (unstructured EHR)	Early fusion	Tree-based multivariable extreme gradient boosting algorithm	Aggregate model in internal test set (HFpEF vs. HFrEF/non-HF): AUROC 90 (88–92), average precision 74
Liu Yi et al. (53)	C	Binary classification task: HF and mild CVD (control) patients	- Imaging features (second lead ECG, CXR) - Tabular features (structured text data)	Early and late (score) fusion	Feature extraction involved using a 1D CNN for ECG signals, DenseNet for chest x-rays, and a three-layer fully connected network for structured text.	The full multimodal ML model using feature level fusion after data augmentation achieved accuracy of 99.92
Botros Jad et al. (57)	C	Binary classification task: HF and healthy/patients with other diseases (control)	- Imaging features (12-lead diagnostic ECG) - Tabular features (blood test results)	Early, middle, and late fusion	A CNN model is utilised for ECG classification, while an XGB algorithm analyses blood test results	The model with late fusion approach achieved accuracy of 97.46, recall of 97.16, specificity of 97.67
Postiglione Marco et al. (54)	C	Multiple binary classification task: positive case (HF) and negative case (nine other diseases)	- Tabular features (raw tabular data, structured data in EHR) - Text features (unstructured data in EHR-Italian clinical notes)	Early and late fusion	For extracting unstructured features, the authors employed a transformer-based model trained on an expert-annotated dataset. This model automatically determined the assertion status—classifying mentions of predefined concepts within clinical notes as “present”, “absent”, or “familiarity”.	Authors only reported the best training configuration and performance per disease. For HF, the best model was logistic regression with synthetic minority over-sampling technique using only unstructured features. It achieved F1 score of 56.2, accuracy of 81.7, precision of 58.6, recall of 60.3
					Authors utilised and compared various ML models: Random Forest, Logistic Regression, SVM, Gaussian Naïve Bayes, Multi-layer perceptron	
Shiraga Takeru et al. (55)	C	Binary classification task: severity or not (separately trained for each disease: aortic valve stenosis, mitral valve regurgitation, left ventricular dysfunction with ejection fractions ≤ 40%)	- Sound features (PCG) - Imaging features (12-lead ECG)	Late fusion	A sequence of ten convolutional layers formed the core of the model. Within this structure, both the first and the last convolutional blocks were succeeded by a ReLU activation function and batch normalization. Max-pooling operations were performed following the 4th, 6th, 9th, and 10th convolutional blocks. This was then followed by a global pooling layer, with a SoftMax function activating the final layer.	Model with XGB using PCG3ch and ECG2pt achieved AUROC of 90.6 (89.96–91.24), F1 score of 43.6 (41.88–45.32), specificity of 87.3 (86.86–87.74), recall of 78.7 (76.48–80.92).
Hardy-Werbin Max et al. (56)	C	Multi-class classification task: COVID-19, HF and Non-COVID Pneumonia and healthy patients.	- abular features (blood test) - Imaging features (CXR)	Late fusion (gradient blending)	MultiCOVID model consisted of an ensemble of 5 distinguished joint models predictions using hard voting to classify the CXR-Blood test pairs	For HF prediction, multiCOVID model achieved AUROC of 92.4 (92.38–92.42), F1 score of 60.3 (60.16–60.44), accuracy of 84 (83.97–84.03), precision of 61.0 (60.86–61.14), recall of 59.6 (59.45–59.75)
Ketabi Sara et al. (61)	C	Multi-class classification task: pneumonia, congestive heart failure, normal	- Imaging features (CXR) - Text features (radiology report including eye-gaze information)	Late fusion	− CNN-based classification models − Chest x-ray: CNN EfficientNet-b0 was encoder − Radiology Report Text: Word2Vec Skip-gram model created sentence embeddings − Later authors applied the model-generated attention maps to visually pinpoint key areas on the input chest x-ray images considered significant for the final diagnostic outcome.	Model with CXR and Report Indication gave the highest performance for congestive HF, AUROC of 96.2 (95.99–96.41)
Zhang Shenghan et al. (62)	C	Binary classification task: HF and negative samples	- Tabular features (EHR structured data) - Text features (clinical note)	Late fusion	Regarding EHR structured data, a deep ensemble methodology was designed, which utilised a multi-layer perceptron to convert sparse tabular data into a compact dense embedding.	The model with ensemble learning achieved AUROC of 99 (98.99–99.01), F1 score of 99 (98.98–99.01), Precision of 99 (98.98–99.01), Recall of 99 (98.98–99.01)
					Regarding text features, authors employed a text filter to isolate important sentences featuring disease-specific terms or keywords; these were subsequently passed first to BlueBERT and then to a TextCNN for further processing.	
Lu Yuan et al. (51)	C	Binary classification task: HF and normal	- Imaging features (CXR) - Text features (Radiology reports)	Early fusion	A model consisted of ResNet-152 extracting features from CXRs, mapped dense multimodal features to the embedding space of pretrained BioBERT, and employed a single-stream network using BioBERT encoder to achieve multimodal fusion	The proposed Res-BioBERT model achieved AUROC of 95.98, AUPRC of 94.38, F1 score of 88.113, Accuracy of 89.02, Precision of 84.319, Specificity of 86.459, Recall of 92.264
Lee Chih-Kuo et al. (64)	C	Binary classification task: NT-proBNP > 300 ng/L (positive) or not	- Imaging features (CXR) - Tabular features (EHR)	Early, middle, and late fusion	There were 2 models including feature extractor and classifier for each type of modality. They used a multilayer perceptron as the feature encoder for EHRs and DenseNet121 to extract features from CXRs and Region of Interests (referring to specific areas within the CXR).	Highest performance model, based on AUROC metric, was multimodal ML model using late fusion. It achieved AUROC of 88.61 (88.54–88.68), Accuracy of 79.51 (79.11–79.91), Specificity 77.31 (75.89–78.73), Recall of 80.38 (79.37–81.39

a Best-performing model metrics with 95% CI where available. All metrics derived from internal holdout test sets unless otherwise specified.

AUPRC, Area under the precision-recall curve; AUROC, Area under the receiver operating characteristic curve; C, Classification objective; CI, Confidence interval; CNN, Convolutional neural network; CXR, Chest x-ray; ECG, Electrocardiogram; EHR, Electronic health record; HRV, Heart rate variability; P, Prognosis objective; PCG, Phonocardiogram.

### 3.5 Performance

**Figure 4:**
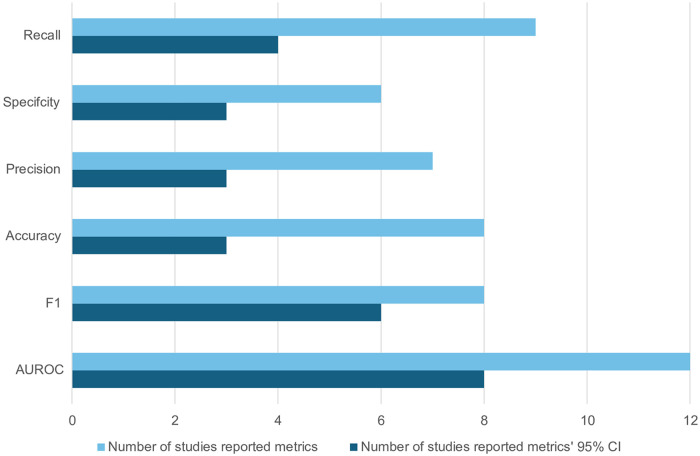
Number of studies reporting performance metrics and their 95% Cis.

Across the 15 reviewed studies, a majority (12 studies) reported the area under the receiver operating characteristic curve (AUROC) metric. Among them, only two-thirds included 95% confidence intervals (CIs). Similarly, eight studies presented F1 scores, with six studies providing 95% CIs. For other performance metrics, Figure 4 illustrates that half or fewer of the reporting studies included 95% CIs.

**Figure 5:**
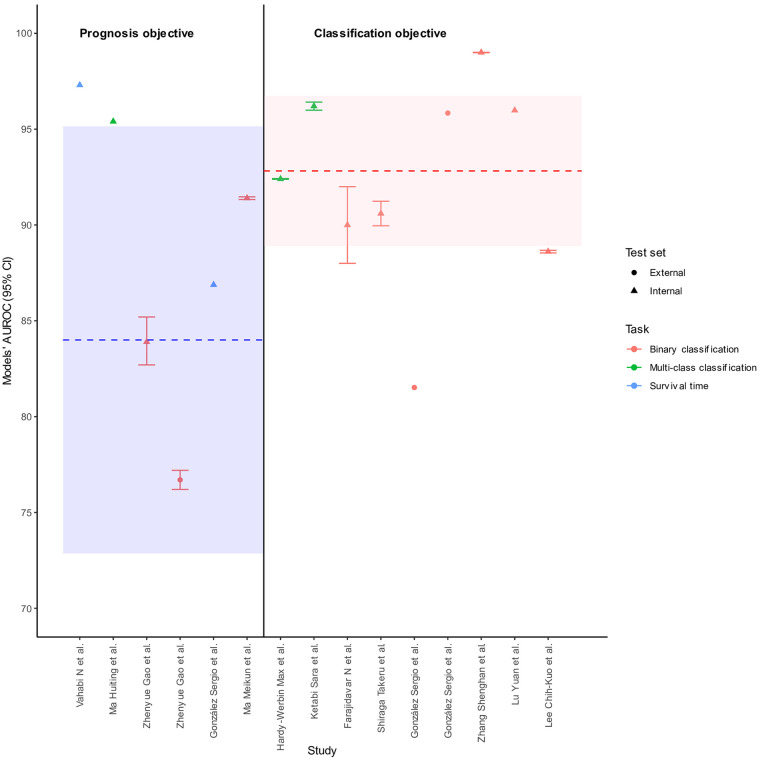
Models' AUROC in 12 studies separated by objectives. Dash line and shaded area mean pooled AUROC and its 95% confidence interval.

Figure 5 displays study-specific AUROC estimates with reported 95% CI in limit, demonstrating moderate to high accuracy across included studies. The pooled AUROC was generated using a random-effect model among studies sharing the same binary classification task and separately for each objective. We leveraged the results from two studies with multi-class classification tasks to calculated pooled AUROC for classification objective as they reported AUROC per class. The pooled AUROC from these studies was 92.83 (95% CI 88.91–96.73, *p* < 0.001) indicating that on average, the models classified heart failure better than guessing. Other statistics (*I*^2^ = 100%, *p*-value < 0.001) indicated these studies do not all share a common underlying effect. The pooled AUROC from prognosis objective studies was 84.00 (95% CI 72.86–95.14, *p* < 0.001), with significant heterogeneity observed (*I*^2^ = 99.94%, *p*-value < 0.001).

Similarly, other reported metrics also indicated strong performance in specific contexts. F1 scores implied robust performance across multiple studies, frequently exceeding 85%–90%. The accuracy often ranged from 85% to 99% in several models (51, 53, 57, 58, 63).

Compared with unimodal models, multimodal approaches generally demonstrate superior performance (52–55, 60, 61, 64). However, one study reported that the superior model for classifying HF (positive class) versus nine other diseases (negative class) was unimodal logistic regression with the synthetic minority oversampling technique, an algorithm that generates synthetic instances for the minority class by interpolating between existing instances. This unimodal model employed a transformer-based model trained on an expert-annotated dataset to automatically determine the assertion status and classify mentions of predefined concepts within clinical notes. Utilising exclusively unstructured features as the inputs, this model achieved an F1 score of 56.2 (54).

### 3.6 Clinical utility

Addressing the inherent opacity of complex ML algorithms is essential for clinical adoption, as practitioners require an understanding of prediction rationales. Seven studies incorporated explainability methods such as SHAP analyses and class activation mapping heatmaps to provide interpretable reasoning (52, 56, 58–61, 64). One study compared the model performance with expert chest radiologists in classifying COVID, non-COVID pneumonia, HF, and healthy participants (56). The model's sensitivity was similar to the mutually agreed prediction of five radiologists, but it showed higher accuracy (69.6% versus 59.3%, *p* < 0.001) (56).

Additionally, two studies demonstrated real-world evaluations of their models. One study validated their model on an independent patient cohort from the same hospital where the training population visited (55). The validation showed the model's inferior performance, particularly for the reduced ejection fraction detection models. Another study translated their research into practical application through the myHeartScore app for cardiovascular disease (CVD) risk assessment, which incorporated ECG interpretations with human-readable features, demonstrating potential for direct clinical implementation (60).

Furthermore, these multimodal ML models utilise inputs familiar to clinicians, such as EHR, ECG, or chest x-ray data (Table 3), closely resembling the diverse data sources that clinicians employ in patient evaluation. Therefore, it facilitates the integration of multimodal learning with established clinical decision-making processes.

## 4 Discussion

Our systematic review demonstrates that multimodal ML approaches broadly outperform unimodal models in HF detection and prognosis prediction. Across the 15 reviewed studies, multimodal models achieved high performance metrics, with AUROC values frequently exceeding 80% and reaching as high as 98% in some cases. Similarly high F1 scores, often exceeding 85%–90%, were reported across multiple studies. The integration of diverse data modalities—primarily combinations of tabular-image, tabular-text, and text-image—enables these models to capture potentially complementary information from different perspectives, enhancing their diagnostic and prognostic capabilities for HF patients.

Our findings align with and extend previous systematic reviews examining ML applications in CVD. The moderate to high AUROC performance in their analysis bears similarity to our findings, where the included studies consistently achieved AUROC values exceeding 85% (65, 66), indicating the great potential of ML in HF classification and prognosis prediction. Notably, neither of these previous reviews specifically distinguished between unimodal and multimodal methodological approaches. While single-modality data processing can reveal patterns imperceptible to human clinicians, the integration of multiple data modalities potentially uncovers previously unrecognised relationships (10). Our review specifically highlights the added value of multimodal fusion strategies. The observed tendency for late fusion to outperform other methods among studies that compared different fusion techniques might be explained not only by the advantages of allowing modality-specific feature extraction before integration, but also by pragmatic considerations. The challenges lay in developing uniform encoding methods suitable for early or middle fusion. Nevertheless, this paradigm is being redefined as models like transformers offer more sophisticated pathways for effective early and middle fusion.

This study identified a substantial deficiency in the incorporation of genetic or ‘omics’ information alongside conventional data modalities in HF prediction frameworks. Only one out of 15 studies leveraged comprehensive genetic modalities, limiting the potential of multimodal approaches to uncover underlying biological insights and subtypes of HF. This deficiency may leave potentially clinical patterns undiscovered, especially in populations with unique phenotypic or genotypic characteristics. Emerging evidence suggests significant potential for personalised genomic risk calculation to increase cardiovascular risk stratification and illuminate novel pathophysiological mechanisms underlying HF development and progression. Therefore, integration of genetic data would capture the full benefits of multimodal ML in HF care (67, 68).

We documented inconsistent performance metric reporting practices, with only approximately two-thirds of studies providing 95% CIs for AUROC measurements and even fewer reporting uncertainty estimates for alternative metrics, thereby preventing formal quantitative synthesis through meta-analysis. Similarly, a study reviewing artificial intelligence for atrial fibrillation prediction (via electrocardiographic data) encountered the same challenges (65). Their analysis of 12 investigations revealed universal reporting of accuracy, but substantial gaps existed in other critical metrics: 16.7% failed to document sensitivity and specificity, 75% omitted F1 scores, and 58.3% lacked AUROC values. This inconsistency persists despite the establishment of specialised reporting frameworks for medical artificial intelligence dating back to 2009 (69), including domain-specific guidelines such as Pineau's reproducibility checklist (70) and the Recommendations for Reporting Machine Learning Analyses in Clinical Research (71). Our review confirms inadequate guideline adherence across the field, which partly contributed to the limited translation of the findings to clinical application. Future investigations employing ML methodologies in cardiovascular medicine should implement recognised reporting standards, including the recently published TRIPOD-AI framework recommended by the EQUATOR Network (https://www.equator-network.org/library) for prediction model development and validation studies (72).

Our systematic examination reveals significant methodological concerns, including the absence of prospective investigational designs and severely limited external validation efforts. Most included studies utilised predominantly Western populations and databases (particularly MIMIC), raising concerns about generalisability to diverse global settings. The clinical relevance of these architectures may be prone to hypothesis-testing rather than clinical decision-supporting. Two included studies were experienced substantial performance deterioration in identifying the reduced ejection fraction (59, 60) when evaluating model performance using independent cohorts, suggesting that results may not extrapolate seamlessly to different clinical environments. This observation aligns with previous research examining ML applications in HF subtyping and risk prediction, where external validation was conducted in only 8.2% of studies and prospective designs were implemented in just 16.5% (73). Nevertheless, prospective evaluations constitute an essential hurdle for ML algorithms aspiring to clinical implementation, as they must demonstrate robustness against real-world challenges (74). To address this, future research must prioritize prospective designs, external validation in varied, real-world populations, and adaptation of federated learning to strengthen clinical impact and reduce health disparities when applying research results into clinical practice. Meanwhile, the clinical implementation of multimodal ML in HF should proceed cautiously, pending further high-quality confirmatory evidence.

Despite the use of advanced architectures, explainability and interpretability are only superficially addressed. Just over half the studies incorporated tools such as SHAP values, class activation maps, or direct comparisons to clinician decision-making. The opacity of “black-box” models presents a barrier to clinician trust and acceptance. However, even achieving high discriminative performance (such as AUROC or F1 score) does not guarantee clinical utility. Because clinical adoption requires not only interpretable predictions but also demonstrating actionable value beyond existing assessment methods, with adequate calibration across decision thresholds and cost-effectiveness analyses—none of which were systematically evaluated in the reviewed studies. Greater emphasis on transparent, interpretable models—through visualizations, rationales for predictions, and clinical validation against expert judgment—is critical for safe and effective clinical deployment.

Our systematic review has several strengths. First, we conducted a comprehensive assessment of model architectures, data sources, and fusion strategies, providing valuable information for future studies and clinical implementation. Second, to reduce the potential for publication bias, our approach involved broad inclusion criteria that covered a range of publication types, such as conference proceedings and preprints. However, in the final analysis, no preprints met all the specified conditions for inclusion. In addition to our strengths, several limitations must be acknowledged. First, conventional publication bias likely influences our findings, as statistically significant results typically receive preferential publication consideration (although formal publication bias assessment was not conducted), and linguistic bias may exist owing to our English‒language restriction. Second, we deliberately employed an inclusive HF definition, acknowledging the substantial heterogeneity in diagnostic criteria across the clinical literature, practice guidelines, and healthcare settings, reflecting the complex pathophysiology of this condition (4). This inclusive approach was strategically selected to capture the broadest possible representation of ML applications in contemporary HF research, despite resulting evaluation metric heterogeneity across the included studies. Third, a significant consideration in interpreting our findings is the sample size distribution across the included literature. We acknowledge that 60% of the included studies utilized cohorts of fewer than 1,000 participants, considering as small studies. This often reflects the complexity of multimodal integration and scarcity in medical image datasets. While this concern can increase susceptibility to optimistic bias, it is important to note that the studies in this subset utilised k-fold cross-validation to enhance the robustness of their performance metrics (52–55, 60–64). Fourth, our pooled AUROC should be interpreted with caution and not used as a universal benchmark. This value represents the upper bound of machine learning capabilities across diverse studies, not expected performance in any specific clinical setting, particularly given the substantial methodological and population heterogeneity observed among the included studies. Fifth, we could not compare the computational complexity and scalability of all included multimodal models as only one study (64) fully reported its computational complexity showing its good potential for scalability (detailed at Supplement Material S3). However, another study translated their multimodal model into a mobile application for cardiovascular risk assessment suggesting the computational efficiency compatible with mobile platform. Still, researchers should consider the trade-offs between model complexity and scalability, particularly for resource-constrained healthcare environments where real-time predictions are required.

We implemented a holistic analysis of the current landscape of multimodal ML approaches for HF classification and prognosis prediction. Our findings highlight the significant potential of multimodal algorithms and their current limitations. Multimodal approaches demonstrate promising performance, suggesting their capability to identify complex patterns across diverse data sources that may enhance HF management. However, critical gaps persist in methodological rigour, including inconsistent performance metric reporting, limited external validation, and the near absence of prospective studies and real-world clinical implementations.

## Data Availability

The original contributions presented in the study are included in the article/Supplementary Material, further inquiries can be directed to the corresponding author.
