# Correction: A systematic review of multimodal machine learning models for heart failure classification and prognosis prediction

**DOI:** 10.3389/fcvm.2026.1863542

**Published:** 2026-05-07

**Authors:** Manh Thang Hoang, Yang Chen, Gregory Slabaugh, Nay Aung

**Affiliations:** 1 William Harvey Research Institute, Queen Mary University of London, London, United Kingdom; 2 Digital Environment Research Institute (DERI), Queen Mary University of London, London, United Kingdom; 3 NIHR Barts Cardiovascular Biomedical Research Centre, Queen Mary University of London, London, United Kingdom; 4 Barts Heart Centre, Barts Health NHS Trust, London, United Kingdom

**Keywords:** heart failure, machine learning, meta analysis, multimodal, review

The order of authors in the author list of the published paper was erroneously given as:

Manh Thang Hoang, Nay Aung, Yang Chen and Gregory Slabaugh

The correct author list reads:

Manh Thang Hoang, Yang Chen, Gregory Slabaugh and Nay Aung.

Four electronic databases, the Web of Science Core Collection, Embase, PubMed, and the IEEE Xplore Cochrane Library, were searched by a reviewer (M.T.).

A correction has been made to the section 2 Materials, 2.1 Search strategy:

“Four electronic databases, the Web of Science Core Collection, Embase, PubMed, and the IEEE Xplore Library, were searched by a reviewer (M.T.).”

The original version of this article has been updated.

