# Supplementary material for: A systematic review of multimodal machine learning models for heart failure classification and prognosis prediction
Source: Front Cardiovasc Med. 2026 Apr 15;13:1770869. doi: 10.3389/fcvm.2026.1770869 (PMC13126653; doi:10.3389/fcvm.2026.1770869)
Supplement: Supplementary file 1 [file Datasheet1.docx]

**SUPPLEMENTS**

Supplement 1: Keywords and search strategy across databases

| **Name of Database** | **Keywords and search strategy** |
| --- | --- |
| Pubmed | #1: ((((((("heart failure") OR ("cardiac failure")) OR ("CHF")) OR ("congestive heart failure")) OR ("left ventricular failure")) OR ("left ventricular dysfunction")) OR ("CCF")) OR ("congestive cardiac failure") |
|  | #2: ((("machine learning") OR ("deep learning")) OR ("artificial intelligence")) OR ("neural network") |
|  | #3: ((((("multimodal") OR ("multi-modal")) OR ("multimodality")) OR ("data fusion")) OR ("multiple data")) OR ("heterogeneous data") |
|  | #4: (((("classification") OR ("prediction")) OR ("prognosis")) OR ("risk stratification")) OR ("diagnosis") |
|  | #1 AND #2 AND #3 AND #4 Filters: from 2014/11/07 - 2024/11/07 |
| Web of Science | #1: (((((((ALL=("heart failure")) OR ALL=("cardiac failure")) OR ALL=("CHF")) OR ALL=("congestive heart failure")) OR ALL=("left ventricular failure")) OR ALL=("left ventricular dysfunction")) OR ALL=("CCF")) OR ALL=("congestive cardiac failure") Timespan: 2014-11-07 to 2024-11-07 (Publication Date) |
|  | #2: (((ALL=("machine learning")) OR ALL=("deep learning")) OR ALL=("artificial intelligence")) OR ALL=("neural network")  Timespan: 2014-11-07 to 2024-11-07 (Publication Date) |
|  | #3: (((((ALL=("multimodal")) OR ALL=("multi-modal")) OR ALL=("multimodality")) OR ALL=("data fusion")) OR ALL=("multiple data")) OR ALL=("heterogeneous data")  Timespan: 2014-11-07 to 2024-11-07 (Publication Date) |
|  | #4: ((((ALL=("classification")) OR ALL=("prediction")) OR ALL=("prognosis")) OR ALL=("risk stratification")) OR ALL=("diagnosis")  Timespan: 2014-11-07 to 2024-11-07 (Publication Date) |
|  | #1 AND #2 AND #3 AND #4 Timespan: 2014-11-07 to 2024-11-07 (Publication Date) |
| IEEE | (((((All Metadata:"heart failure") OR (All Metadata:"cardiac failure") OR (All Metadata:"CHF") OR (All Metadata:"congestive heart failure") OR (All Metadata:"left ventricular failure") OR (All Metadata:"left ventricular dysfunction") OR (All Metadata:"CCF") OR (All Metadata:"congestive cardiac failure") refined by:Search Latest Date:11/07/2014-11/07/2024 )) AND ((All Metadata:"machine learning") OR (All Metadata:"deep learning") OR (All Metadata:"artificial intelligence") OR (All Metadata:"neural network") refined by:Search Latest Date:11/07/2014-11/07/2024 )) AND ((All Metadata:"multimodal") OR (All Metadata:"multi-modal") OR (All Metadata:"multimodality") OR (All Metadata:"data fusion") OR (All Metadata:"multiple data") OR (All Metadata:"heterogeneous data") refined by:Search Latest Date:11/07/2014-11/07/2024 )) AND ((All Metadata:"classification") OR (All Metadata:"prediction") OR (All Metadata:"prognosis") OR (All Metadata:"risk stratification") OR (All Metadata:"diagnosis") refined by:Search Latest Date:11/07/2014-11/07/2024 ) |
| Embase | #1: 'heart failure'/exp OR 'heart failure' OR 'cardiac failure'/exp OR 'cardiac failure' OR 'chf' OR 'congestive heart failure'/exp OR 'congestive heart failure' OR 'left ventricular failure'/exp OR 'left ventricular failure' OR 'left ventricular dysfunction'/exp OR 'left ventricular dysfunction' OR 'ccf' OR 'congestive cardiac failure'/exp OR 'congestive cardiac failure' |
|  | #2: 'machine learning'/exp OR 'machine learning' OR 'deep learning'/exp OR 'deep learning' OR 'artificial intelligence'/exp OR 'artificial intelligence' OR 'neural network'/exp OR 'neural network' |
|  | #3: 'multimodal' OR 'multi-modal' OR 'multimodality' OR 'data fusion'/exp OR 'data fusion' OR 'multiple data' OR 'heterogeneous data' |
|  | #4: 'classification'/exp OR 'classification' OR 'prediction'/exp OR 'prediction' OR 'prognosis'/exp OR 'prognosis' OR 'risk stratification'/exp OR 'risk stratification' OR 'diagnosis'/exp OR 'diagnosis' |
|  | #1 AND #2 AND #3 AND #4 AND [07-11-2014]/sd NOT [08-11-2024]/sd |

Supplement 2: Details of studies’ quality assessment based on QUADAS-2 and QUAPAS

| Study no. | Objective | QUADAS-2 and QUAPAS | | | | | | | | |
| --- | --- | --- | --- | --- | --- | --- | --- | --- | --- | --- |
|  |  | Risk of bias | | | | | Applicability Concerns | | | |
|  |  | Patient Selection / **Participants** | Index Test | Reference Standard/ **Outcome** | Flow and Timing | **Analysis** | Patient Selection / **Participants** | Index Test | Reference Standard/ **Outcome** | **Flow and Timing** |
| 1 | P | Unclear | Unclear | Low | Low | Low | Low | Low | Low | Low |
| 2 | C | Low | Low | Low | High |  | Low | Low | Low |  |
| 3 | C | Unclear | Low | Unclear | Low |  | Unclear | Low | Low |  |
| 4 | C | Unclear | Low | Unclear | Low |  | Unclear | Low | Low |  |
| 5 | C | Low | Low | Low | Low |  | Low | Low | Low |  |
| 6 | P | Unclear | Unclear | Low | Low | High | Low | Low | Low | Low |
| 7 | P | Low | Unclear | Low | Low | Unclear | Low | Low | Low | Low |
| 8 | C | Unclear | Low | Low | Low |  | Low | Low | Low |  |
| 9 | C | Unclear | Low | Low | Low |  | Low | Low | Low |  |
| 10 | P, C | Unclear | Unclear | Low | Low | High | Low | Low | Low | Low |
| 11 | C | Unclear | Low | Low | Low |  | Low | Low | Low |  |
| 12 | C | Low | Low | Low | Low |  | Low | Low | Low |  |
| 13 | P | Unclear | Unclear | Low | Low | Low | Low | Low | Low | Low |
| 14 | C | Unclear | Low | Low | Low |  | Unclear | Low | Low |  |
| 15 | C | Unclear | Low | Low | Low |  | Low | Low | Low |  |

*C = Classification objective, P = Prognosis objective*

*QUADAS-2’s domains include Patient Selection, Index Test, Reference Standard, Flow and Timing in Risk of Bias and Patient Selection, Index Test, Reference Standard in Applicability Concerns*

*QUAPAS’s domains include Participants, Index Test, Outcome, Flow and Timing, Analysis in Risk of Bias, Participants, Index Test, Outcome, Flow and Timing in Applicability Concerns*

List of studies under Study no. column

1 Vahabi, N., et al., Cox-sMBPLS: An Algorithm for Disease Survival Prediction and Multi-Omics Module Discovery Incorporating Cis-Regulatory Quantitative Effects. Front Genet, 2021. 12: p. 701405.

2 Farajidavar, N., et al., Diagnostic signature for heart failure with preserved ejection fraction (HFpEF): a machine learning approach using multi-modality electronic health record data. BMC Cardiovasc Disord, 2022. 22(1): p. 567.

3 Liu, Y., et al., Enhancing heart failure diagnosis through multi-modal data integration and deep learning. Multimedia Tools and Applications, 2023.

4 Botros, J., F. Mourad-Chehade, and D. Laplanche, Explainable multimodal data fusion framework for heart failure detection: Integrating CNN and XGBoost. Biomedical Signal Processing and Control, 2025. 100.

5 Postiglione, M., et al., Harnessing Multi-modality and Expert Knowledge for Adverse Events Prediction in Clinical Notes. Image Analysis and Processing - Iciap 2023 Workshops, Pt Ii, 2024. 14366: p. 119-130.

6 Ma, H.T., et al., Heart failure Let's start with the MDL-HFP model. Information Systems, 2024. 125.

7 Gao, Z., et al., Improving the Prognostic Evaluation Precision of Hospital Outcomes for Heart Failure Using Admission Notes and Clinical Tabular Data: Multimodal Deep Learning Model. J Med Internet Res, 2024. 26: p. e54363.

8 Shiraga, T., et al., Improving Valvular Pathologies and Ventricular Dysfunction Diagnostic Efficiency Using Combined Auscultation and Electrocardiography Data: A Multimodal AI Approach. Sensors (Basel), 2023. 23(24).

9 Hardy-Werbin, M., et al., MultiCOVID: a multi modal deep learning approach for COVID-19 diagnosis. Sci Rep, 2023. 13(1): p. 18761.

10 González, S., et al., Multi-modal heart failure risk estimation based on short ECG and sampled long-term HRV. Information Fusion, 2024. 107.

11 Ketabi, S., et al., Multimodal Learning for Improving Performance and Explainability of Chest X-Ray Classification. Medical Image Computing and Computer Assisted Intervention, Miccai 2023 Workshops, 2023. 14393: p. 107-116.

12 Zhang, S., et al. PheME: A deep ensemble framework for improving phenotype prediction from multi-modal data. in 2023 IEEE 11th International Conference on Healthcare Informatics (ICHI). 2023.

13 Ma, M., et al., Predicting heart failure in-hospital mortality by integrating longitudinal and category data in electronic health records. Med Biol Eng Comput, 2023. 61(7): p. 1857-1873.

14 Lu, Y., C. Zhang, and F. Tang. ResBioBERT: Deep learning combined with multimodal data for heart failure diagnosis. in 2024 16th International Conference on Intelligent Human-Machine Systems and Cybernetics (IHMSC). 2024.

15 Lee, C.K., et al., Multimodal deep learning models utilizing chest X-ray and electronic health record data for predictive screening of acute heart failure in emergency department. Comput Methods Programs Biomed, 2024. 255: p. 108357.

Supplement 3: Computational complexity reported

| Study no. | Computational complexity reported status* | Reported metrics / Note |
| --- | --- | --- |
| 1 | No |  |
| 2 | No |  |
| 3 | No | The study reported using a server equipped with NVIDIA GeForce RTX3060 GPU |
| 4 | Partially | The study only reported the prediction time. Reducing the number of blood test input features from 31 to 3, multimodal machine learning model with late fusion approach (the best performance model) recorded the prediction time on the test set decreasing from 0.013s to 0.005s |
| 5 | No |  |
| 6 | No | The study only reported training settings (50 epochs, batch size 32, ...), hardware environment (CPU Inter (R) Xeon (R) CPU of 3.66 GHZ, Memory 16 G, GPU NVIDIA Tesla V100, 32 G), and software environment (CentOS 7.0, Pytorch=1.7.1) but not the runtime or computational burden. |
| 7 | No |  |
| 8 | No |  |
| 9 | No | In the supplement, the study only reported hardware environment (a CentOs7 server with 4 Nvidia RTX2080Ti GPUs, running CUDA version 10.0) |
| 10 | No |  |
| 11 | No |  |
| 12 | No |  |
| 13 | No |  |
| 14 | No |  |
| 15 | Yes | The study reported as following “For the Tesla P100-PCIE-16GB, the single-precision computing performance was 9.3 Tflops and the double-precision computing performance was 4.7 Tflops. When using single-precision, preprocessing took 6.81 seconds with a CPU memory usage of 23.66 GB, while inference took 3.67 seconds with a CPU memory usage of 23.62 GB. When using double-precision, preprocessing took 7.16 seconds with a CPU memory usage of 24.27 GB, while inference took 4.62 seconds with a CPU memory usage of 24.21 GB. In both single-precision and double-precision, we obtained the same results and the accuracy was 0.75 for the four cases in the experiments.  For the NVIDIA RTX A6000, the single-precision computing performance was 38.7 TFLOPS. When using single-precision, preprocessing takes 6.86 seconds with a CPU memory usage of 33.19 GB, while inference took 4.95 seconds with a CPU memory usage of 33.00 GB. When using double-precision, preprocessing took 7.36 seconds with a CPU memory usage of 33.63 GB, while inference ta took kes 6.49 seconds with a CPU memory usage of 33.54 GB. In both single-precision and double-precision, we obtained the same results and the accuracy was 0.75 for the four cases in the experiments.  For the NVIDIA TITAN V, the single-precision computing performance was 12.29 Tflops and the double-precision computing performance is 6.14 Tflops. When using single-precision, preprocessing took 8.38 seconds with a CPU memory usage of 21.33 GB, while inference took 3.79 seconds with a CPU memory usage of 21.39 GB. When using double-precision, preprocessing took 9.25 seconds with a CPU memory usage of 23.26 GB, while inference took 4.86 seconds with a CPU memory usage of 21.43 GB. For both single-precision and double-precision, we obtained the same results and the accuracy was 0.75 for the four cases in the experiments.” |

** Computational complexity is considered reported if the paper includes training time (per epoch or total), prediction time, model size, FLOPs, memory usage, efficiency trade-offs (despite adding multiple modalities)*

List of studies under Study no. column is the same as Supplement 2
